# The Sterilization of Water by Heat

**Published:** 1892-11

**Authors:** 


### THE STERILIZATION OF WATER BY HEAT.

THE very great necessity of rendering water sterile, particularly during the prevalence of infectious diseases, is becoming more and more recognized as a method of preventive treatment.

In order to meet this need, there has been manufactured in France a portable boiler, with suitable containers attached, which can be taken by horse-power from house to house, providing the populace with water which has been sterilized by heat, and which will remain with its supplying-tube drawing water from the river or well which supplies the town, and deliver it to the people who bring their vessels for the daily supply. The advantages of such a method cannot be overestimated. The *Scientific American*, in its issue for July 9, 1892, publishes a picture of the apparatus, and a description which is taken from *La Nature.*

It consists of a boiler with an independent steam reservoir, one or more exchanges, and a filter. The exchanges, which are cylinders provided with worms, constitute the most interesting and original part. The impure cold water that they receive is heated by the temperature of the boiled water circulating in the return worms, and this same boiled water becomes cooled therein by giving up its heat to the water which goes to the boiler. In this way the exchange of temperature is effected without expense, and it is possible to easily furnish, on its exit from the apparatus, water sufficiently cool to be used at once.

In fact, experience has proved that water that has been submitted in this apparatus, for at least fifteen minutes, to a minimum temperature of 120° C., may make its exit therefrom with a temperature but 2° higher than that which it had when it entered. As for the microörganisms that it contained, there no longer remains a trace of them. It is absolutely sterilized.

The method has rendered signal service in the barracks of the Marine Corps at Brest, where typhoid fever occurred for many years in such a way as to be almost epidemic.—*Therapeutic Gazette.*

